# An *in vivo cis*-Regulatory Screen at the Type 2 Diabetes Associated *TCF7L2* Locus Identifies Multiple Tissue-Specific Enhancers

**DOI:** 10.1371/journal.pone.0036501

**Published:** 2012-05-10

**Authors:** Daniel Savic, Graeme I. Bell, Marcelo A. Nobrega

**Affiliations:** 1 Department of Human Genetics, University of Chicago, Chicago, Illinois, United States of America; 2 Department of Medicine, University of Chicago, Chicago, Illinois, United States of America; University of Birmingham, United Kingdom

## Abstract

Genome-wide association studies (GWAS) have repeatedly shown an association between non-coding variants in the *TCF7L2* locus and risk for type 2 diabetes (T2D), implicating a role for *cis*-regulatory variation within this locus in disease etiology. Supporting this hypothesis, we previously localized complex regulatory activity to the *TCF7L2* T2D-associated interval using an *in vivo* bacterial artificial chromosome (BAC) enhancer-trapping reporter strategy. To follow-up on this broad initial survey of the *TCF7L2* regulatory landscape, we performed a fine-mapping enhancer scan using *in vivo* mouse transgenic reporter assays. We functionally interrogated approximately 50% of the sequences within the T2D-associated interval, utilizing sequence conservation within this 92-kb interval to determine the regulatory potential of all evolutionary conserved sequences that exhibited conservation to the non-eutherian mammal opossum. Included in this study was a detailed functional interrogation of sequences spanning both protective and risk alleles of single nucleotide polymorphism (SNP) rs7903146, which has exhibited allele-specific enhancer function in pancreatic beta cells. Using these assays, we identified nine segments regulating various aspects of the *TCF7L2* expression profile and that constitute nearly 70% of the sequences tested. These results highlight the regulatory complexity of this interval and support the notion that a *TCF7L2 cis*-regulatory disruption leads to T2D predisposition.

## Introduction

Intronic variation located in a 92-kb interval within the *Transcription Factor 7-Like 2* (*TCF7L2*) gene locus, a transcriptional regulator of canonical Wnt signaling [1], [2], is the strongest determinant for type 2 diabetes (T2D) susceptibility identified to date [3], [4], [5]. Indeed, associations have been reported in populations from across the globe [4] and variation in this locus remain the strongest genetic determinant of T2D risk in humans [6]. As both *in vitro* and *in vivo* functional analyses support a role for TCF7L2 in glucose metabolism [7], [8], [9], [10], [11], [12], [13], [14], [15], this transcription factor is regarded as the candidate target gene of the association.

The underlying molecular, cellular and physiological mechanism(s) by which TCF7L2 affects T2D risk are largely unknown. The results, to date, point to a role for variation in long-range *cis*-regulatory elements in T2D pathogenesis through alterations in *TCF7L2* expression. In this regard, two independent studies in pancreatic islets uncovered allele-specific enhancer activity for sequences spanning single nucleotide polymorphism (SNP) rs7903146, the variant showing the strongest association to T2D [16], [17]. These analyses are consistent with the recent functional studies of non-coding variants in other disease-associated GWAS loci and highlight the importance of *cis*-regulatory variation in affecting disease risk [18]. From the standpoint of the common disease common variant (CDCV) hypothesis, the implications for common disease risk are clear as variation in these regulatory sequences can lead to a compartmentalization of phenotypic effects as enhancer elements largely govern activity in a spatial and temporal context, mitigating pleiotropic effects [18]. This would ostensibly allow these disadvantageous non-coding variants to reach higher frequencies in human populations as compared with protein-coding variants that can elicit disruptions with broader consequences on target gene activity [18].

We previously interrogated the *cis*-regulatory landscape of *TCF7L2* using a bacterial artificial chromosome (BAC) enhancer-trapping strategy and identified widespread enhancer activity that we localized to the association interval [12]. However, a systematic fine-mapping analysis of the association interval is still lacking. To follow-up on this BAC survey, here we characterized the long-range *cis*-regulatory landscape of this T2D-associated genomic locus through an *in vivo* fine-mapping approach. We demonstrate that the association interval harbors a wide variety of tissue-specific enhancers, including a subset that drives expression in peripheral tissues involved in glucose homeostasis, adding support to a potential regulatory defect in T2D etiology.

## Methods

### Ethics Statement

All mice were housed at the University of Chicago. Veterinary care was available on a 24-hour basis. Mice were monitored daily for any signs of illness or discomfort. All experiments were conducted in strict accordance with institutional rules and approved by the University of Chicago Institutional Animal Care and Use Committee, protocol number 71656 (M.A.N.).

### Molecular cloning

Conserved sequences within the association interval were cloned (Table S1) with Gateway technology (Invitrogen) in a custom vector containing a heat shock minimal promoter (Hsp68) driving *lacZ* expression. The transgenic *lacZ* plasmids were linearized, resuspended in 1x microinjection buffer and used for pronuclear injection into fertilized oocytes (CD-1) using standard protocols approved by the University of Chicago Institutional Animal Care and Use Committee.

### Mouse *in vivo* Transgenic Reporter Assays

Animals were sacrificed using carbon dioxide gas followed by cervical dislocation. Embryos were harvested at embryonic day 15.5 (E15.5) or 16.5 (E16.5). Pancreatic staining was also performed postnatally on day 0 (P0) and 6 (P6). Following harvesting and dissection, embryos and tissues were placed into cold 100 mM phosphate buffer, pH 7.3 (PBS), followed by an hour of incubation with 4% paraformaldehyde at 4°C. Tissues were then washed with 1x PBS and further washed two additional times for 20 min using lacZ wash buffer (2 mM MgCl_2_; 0.01% deoxycholate; 0.02% NP-40; 100 mM phosphate buffer, pH 7.3), and stained for 16–20 hours at room temperature with lacZ staining solution (1 mg/ml X-gal; 4 mM potassium ferrocyanide; 4 mM potassium ferricyanide; 20 mM Tris-HCl, pH 7.5 in wash buffer). After staining, embryos and tissues were rinsed 5 times in PBS and post-fixed and stored in 4% paraformaldehyde at 4°C. Images were taken using a Leica MZ 16 F imaging system.

## Results

### Enhancer fine-mapping at the *TCF7L2* association interval

**Figure 1:**
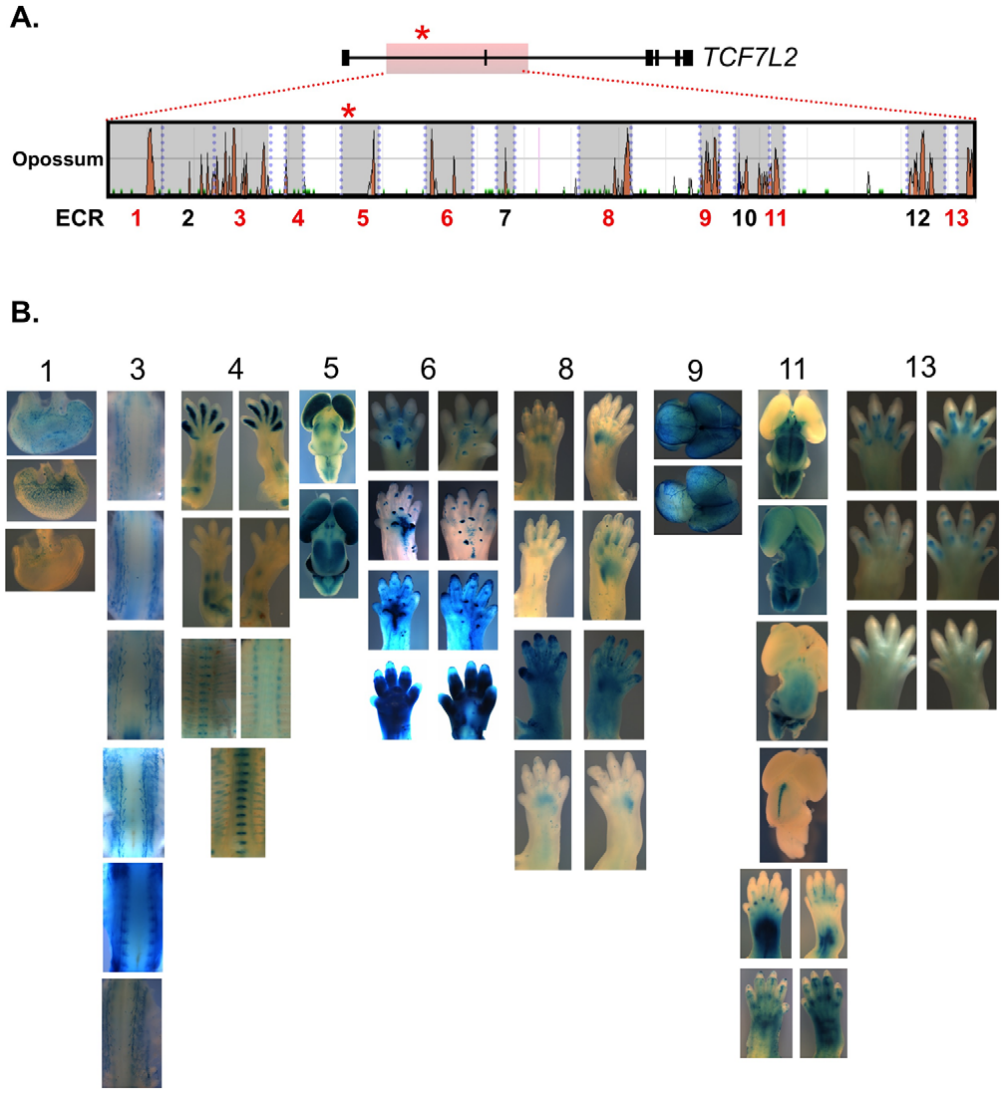
Enhancer screen of evolutionary conserved sequences within *TCF7L2* association interval. (A) The *TCF7L2* gene is shown above with the 92-kb T2D-associated internal highlighted in red. A red asterisk marks SNP rs7903146. Sequence conservation between human-opossum is given (ECR genome browser, [43]). The 13 evolutionary conserved regions (ECRs) harboring conservation down to opossum are highlighted in grey and numbered below. Regions exhibiting reproducible enhancer activity are marked in red. (B) Reproducible expression profiles from ECRs at embryonic day 15.5 (E15.5) are shown. Expression can be seen in the stomach (ECR 1), neurons near the spine (ECR 3), limb bones and axial skeletion (ECR 4), forebrain (dorsal view, ECR 5), walking pads (ECR 6), limb vasculature (ECR 8), brain vasculature (dorsal view, ECR 9), limbs and midbrain (dorsal view, ECR 11), and phalangies (ECR 13). In ECRs 4, 6, 8, 11 and 13, both forelimb (left) and hindlimb (right) images are given. Reproducible regulatory activities were not identified for ECRs 2, 7, 10 and 12.

As sequence conservation is a predictor of function [19], 13 evolutionary conserved regions (ECRs) spanning sequences exhibiting significant conservation between human and the non-eutherian mammal opossum were tested in mouse transgenic assays (Figure 1). These ECRs were cloned into a *lacZ* reporter construct driven by a heat shock protein 68 (Hsp68) minimal promoter. In total ∼48.7 kb of sequence, or approximately 50% of the T2D-associated interval, was tested for regulatory activity *in vivo* (Figure 1A). For each construct, we obtained multiple transgenic lines to ensure reproducibility of enhancer patterns across independent transgenic lines. Of the 13 evolutionarily conserved regions, we observed reproducible enhancer activity in 9/13 (69%) regions tested (ECR 1, 3, 4, 5, 6, 8, 9, 11 and 13).

The enhancers mapping to these intervals exhibit a diverse array of spatial expression patterns that were reproducible across independent transgenic lines (Figure 1B). Interestingly, five regions (ECR 1, 4, 5, 11 and 13) exhibited regulatory potential in tissues with known roles in controlling glucose homeostasis such as the stomach (ECR 1), bone (ECR 4 and 13) and brain (ECR 5 and 11). Beta-galactosidase staining was also localized to spinal neurons (ECR 3), walking pads (ECR 6) and the vasculature of both the limbs (ECR 8) and brain (ECR 9).

### Functional analysis of sequences spanning SNP rs7903146

**Figure 2:**
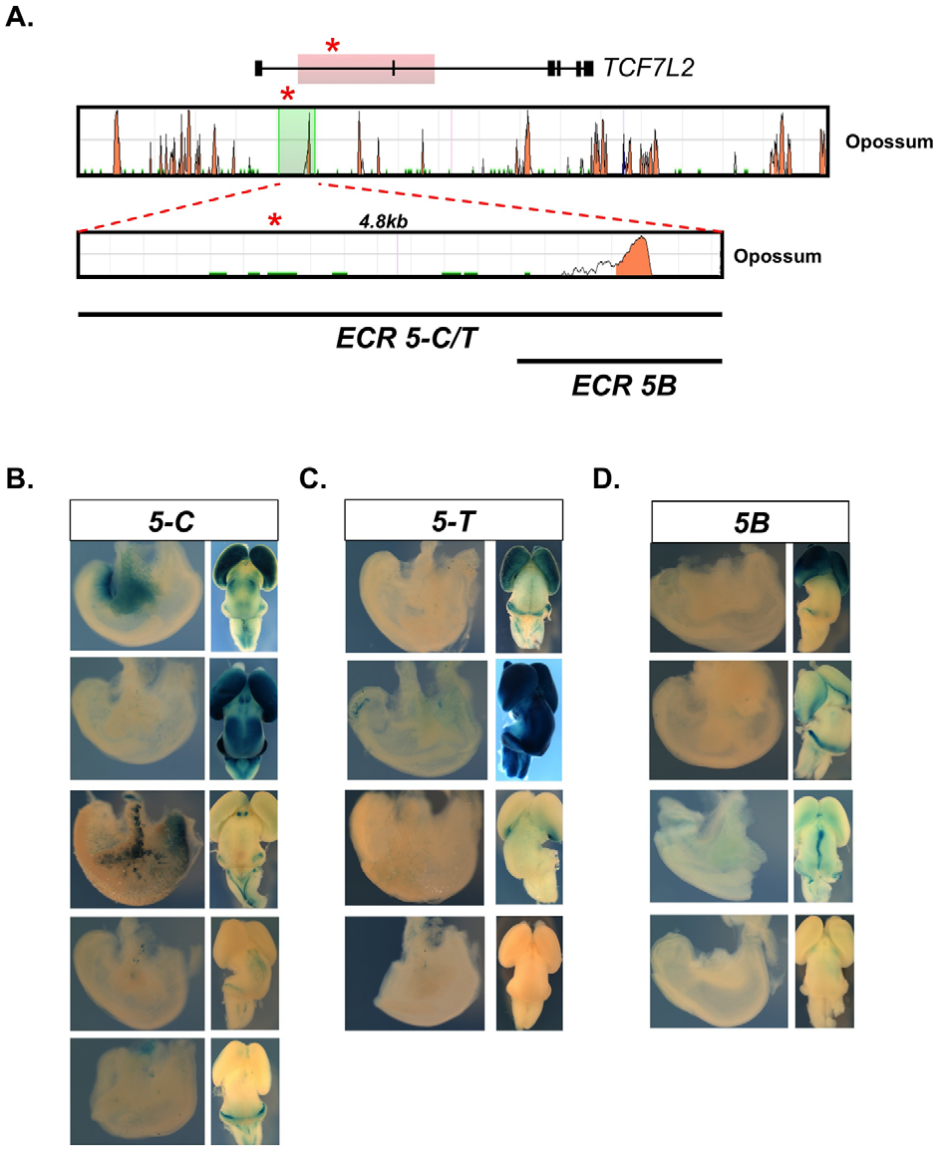
Functional analysis of SNP rs7903146. (A) The association interval within the *TCF7L2* gene locus is highlighted in red above. A red asterisk marks SNP rs7903146. Sequence conservation between human-opossum (ECR genome browser, [43]) is given for the entire association interval while the sequence tested is highlighted in green. Within the tested region, sequence conservation between human-opossum is given below (ECR genome browser). Positions of regions spanning SNP rs7903146 (5-C and 5-T) as well as a shorter sequence limited to the downstream ECR (5B) are shown below. (B)-(D) Images of pancreas (attached to the stomach) (left) and brain (right, dorsal view) obtained from independent transgenic lines (rows) are shown at E15.5 or E16.5 (ECR 5-C row 3 only). (B) and (C) Regions 5-C and 5-T exhibits inconsistent pancreatic staining while maintaining reproducible forebrain expression. (B) Construct 5B harbors forebrain expression but no pancreatic staining.

We next investigated the regulatory potential of sequences containing SNP rs7903146. For this analysis, we utilized the previous results of the construct that encompassed the protective C allele at SNP rs7903146 from ECR 5 (ECR 5-C) and engineered an identical construct that spanned the risk T allele at SNP rs7903146 (ECR 5-T; Figure 2A). In order to dissociate any regulatory effects exhibited by a conserved sequence situated downstream of this SNP, we further generated a shorter construct (ECR 5B) that was restricted to this region of conservation (Figure 2A). As SNP rs7903146 resides within a primate-specific short interspersed nuclear element (SINE) and ‘enhancer boosting’ properties were demonstrated for repetitive elements [20], this approach also allows for the identification of potential ‘enhancer boosting’ activity. All constructs were sequence-verified prior to pronuclei injections. For each of these new constructs, we obtained multiple transgenic lines (Figure 2B, C, D).

All constructs exhibited reproducible expression within the forebrain across multiple transgenic lines, suggesting that the conserved sequence downstream of SNP rs7903146 governs this activity (Figure 2B, C, D). However, we observed no allelic-specific (comparing ECR 5-C and 5-T) or ‘enhancer boosting’ effects (comparing ECR 5B and 5-C/T) as forebrain expression across all constructs was largely consistent. Although some pancreatic expression was present in mice harboring the longer constructs encompassing SNP rs7903146 (ECR 5-C and 5-T) and this pattern was absent in transgenic lines containing the shorter construct ECR 5B, this activity was not highly reproducible across multiple independent transgenic lines, nor did this expression exhibit allelic-specific effects. This conclusion is further corroborated by analyses at postnatal developmental stages in transgenic animals as consistent allelic differences were not observed at postnatal days 0 or 6 (Figure S1).

## Discussion

Our screen defines the fine-scale regulatory landscape of the *TCF7L2* T2D-associated region. To our knowledge, this is the first detailed mapping study conducted on this GWAS-associated region. Importantly, the enhancers identified from this fine-mapping scan recapitulate various aspects of BAC enhancer activities that we previously uncovered at this locus [12]. While significant attention has been given to the role of *TCF7L2* in pancreatic islets, our data delineates several regions harboring regulator activity in peripheral metabolic tissues. In particular, we identified elements that govern expression within the bone, brain and stomach.

The localization of bone enhancers is not surprising, given the well established role of the canonical Wnt signaling in bone formation [21]. For instance, ablation of Wnt regulators leads to bone mass defects in mice [22], [23], [24], [25] while disruptions of Wnt signaling antagonists generates opposing phenotypes [26], [27]. As a previously unappreciated role for bone in the regulation of glucose homeostasis has been recently established, this organ has become an interest to the diabetes community [28], [29]. Consequently, investigations of this transcription factor in bone-mediated glucose metabolism are clearly warranted.

The fine-mapping of a stomach enhancer within the associated interval is of relevance as this tissue secretes the orexigenic hormone ghrelin that is involved in energy and glucose homeostasis [30], [31]. A recent study further uncovered a correlation between a putative *TCF7L2* neuroendocrine splicing variant and the anorexigenic peptide *CART* [32], supporting a role for this canonical Wnt regulator in satiety. The extent of TCF7L2 involvement in energy metabolism and the further implications this may have for T2D risk is a disease mechanism demanding more scrutiny.

The canonical Wnt signaling pathway is also implicated in diverse neurological disorders such as autism, Alzheimer's disease and schizophrenia [33]. Consistent with a neurological function, the *TCF7L2* T2D-associated interval has been implicated in schizophrenia risk [34]. Indeed, we observed behavioral phenotypes in mice with altered Tcf7l2 levels [35]. As the historical co-morbidity between T2D and schizophrenia is well documented, this may point to a common disease etiology [36], [37]. In light of these observations, our identification of several brain enhancers, and in particular a forebrain enhancer situated in the vicinity of SNP rs7903146, may be of interest.

We did not observe robust allelic-specific enhancer activity for SNP rs7903146. Although we cannot exclude potential effects at other developmental stages, the use of a non-native promoter element (Hsp68) or potential complex long-range interactions (i.e enhancer-enhancer) that was not assessed by our assay may explain these results. The previous localization of a pancreatic enhancer using an *in vivo* BAC transgenic strategy supports these conclusions [12]. Alternatively, as the allelic-specific properties at this locus were uncovered through cell-based luciferase and open chromatin assays [16], [17], our results may primarily reflect a limitation in assessing quantitative differences with qualitative approaches. This carries broader implications for GWAS loci in general as potential causal variants are likely to constitute a modest effect on disease risk [38], [39], [40] and therefore may generate finer regulatory defects that are difficult to assess *in vivo*.

Our enhancer screen was also largely restricted to one embryonic developmental stage, E15.5. Despite this limitation, our previous BAC results suggest that the regulatory landscape at this locus is faithfully maintained into adulthood [12]. Another potential concern is the use of evolutionary conserved sequences in our *in vivo* assays. It has become common knowledge that a number of *cis*-regulatory regions, including those involved in embryonic development, lack evolutionary conservation across distant species. Our *in vivo* scan is far from being exhaustive, and we most likely missed other *cis*-regulatory elements embedded in the ∼50% of sequences within the T2D-associated region that we did not test in this study. Nevertheless, our results conclusively indicate that the T2D-associated interval contains at least nine tissue-specific regulatory elements. Despite the strong concordance with the endogenous *TCF7L2* expression profile [12], we cannot definitively rule out the possibility that some of these enhancers could be involved in the regulation of neighboring genes such as the upstream gene *VTI1A*. Genetic variation in a number of these enhancers, common or rare in populations, may lead to alterations in *TCF7L2* or neighboring gene expression, leading to various phenotypic consequences. To that end, the same genomic interval has now also been associated with increased risk to schizophrenia [34], colorectal cancer [41] and coronary artery disease [42].

Our results highlight the complex regulatory nature of the 92-kb T2D-associated region of *TCF7L2* and support the hypothesis that sequence variation within distal *cis*-regulatory elements are mediators of T2D susceptibility. The identification of several enhancers that drive expression in diverse metabolic domains further points to a possible disease etiology involving peripheral metabolic tissues.

## Supporting Information

Figure S1 **Postnatal analyses of pancreatic expression.** Stable transgenic lines were stained for pancreatic beta-galactosidase activity on postnatal days 0 (P0, top panel) and 6 (P6, bottom panel). Pancreatic images for sequences spanning the protective C allele at SNP rs7903146 (5-C) and risk T allele at SNP rs7903146 (5-T) are shown.
(TIFF)

Table S1 **Primer sequences for amplification of evolutionary conserved regions within the TCF7L2 association interval.** The evolutionary conserved region (ECR) is numbered in the first column. Subsequent columns give the primer pair sequences (in 5′ to 3′ orientation) for each ECR.
(TIFF)

## References

[1] Moon RT, Kohn AD, De Ferrari GV, Kaykas A (2004). WNT and beta-catenin signalling: diseases and therapies.. Nat Rev Genet.

[2] Clevers H (2006). Wnt/beta-catenin signaling in development and disease.. Cell.

[3] Grant SF, Thorleifsson G, Reynisdottir I, Benediktsson R, Manolescu A (2006). Variant of transcription factor 7-like 2 (TCF7L2) gene confers risk of type 2 diabetes.. Nat Genet.

[4] Cauchi S, El Achhab Y, Choquet H, Dina C, Krempler F (2007). TCF7L2 is reproducibly associated with type 2 diabetes in various ethnic groups: a global meta-analysis.. J Mol Med.

[5] Lyssenko V (2008). The transcription factor 7-like 2 gene and increased risk of type 2 diabetes: an update.. Curr Opin Clin Nutr Metab Care.

[6] Frayling TM (2007). Genome-wide association studies provide new insights into type 2 diabetes aetiology.. Nat Rev Genet.

[7] Lyssenko V, Lupi R, Marchetti P, Del Guerra S, Orho-Melander M (2007). Mechanisms by which common variants in the TCF7L2 gene increase risk of type 2 diabetes.. J Clin Invest.

[8] Norton L, Fourcaudot M, Abdul-Ghani MA, Winnier D, Mehta FF (2011). Chromatin occupancy of transcription factor 7-like 2 (TCF7L2) and its role in hepatic glucose metabolism.. Diabetologia.

[9] Le Bacquer O, Shu L, Marchand M, Neve B, Paroni F (2011). TCF7L2 splice variants have distinct effects on {beta}-cell turnover and function..

[10] Figeac F, Uzan B, Faro M, Chelali N, Portha B (2010). Neonatal growth and regeneration of beta-cells are regulated by the Wnt/beta-catenin signaling in normal and diabetic rats.. Am J Physiol Endocrinol Metab.

[11] Zhou Y, Zhang E, Berggreen C, Jing X, Osmark P (2011). Survival of pancreatic beta cells is partly controlled by a TCF7L2-p53-p53INP1-dependent pathway..

[12] Savic D, Ye H, Aneas I, Park SY, Bell GI (2011). Alterations in TCF7L2 expression define its role as a key regulator of glucose metabolism..

[13] Ahlzen M, Johansson LE, Cervin C, Tornqvist H, Groop L (2008). Expression of the transcription factor 7-like 2 gene (TCF7L2) in human adipocytes is down regulated by insulin.. Biochem Biophys Res Commun.

[14] Cawthorn WP, Heyd F, Hegyi K, Sethi JK (2007). Tumour necrosis factor-alpha inhibits adipogenesis via a beta-catenin/TCF4(TCF7L2)-dependent pathway.. Cell Death Differ.

[15] Yi F, Brubaker PL, Jin T (2005). TCF-4 mediates cell type-specific regulation of proglucagon gene expression by beta-catenin and glycogen synthase kinase-3beta.. J Biol Chem.

[16] Gaulton KJ, Nammo T, Pasquali L, Simon JM, Giresi PG (2010). A map of open chromatin in human pancreatic islets.. Nat Genet.

[17] Stitzel ML, Sethupathy P, Pearson DS, Chines PS, Song L (2010). Global epigenomic analysis of primary human pancreatic islets provides insights into type 2 diabetes susceptibility loci.. Cell Metab.

[18] Sakabe NJ, Savic D, Nobrega MA (2012). Transcriptional enhancers in development and disease.. Genome Biol.

[19] Boffelli D, Nobrega MA, Rubin EM (2004). Comparative genomics at the vertebrate extremes.. Nat Rev Genet.

[20] Smith AM, Sanchez MJ, Follows GA, Kinston S, Donaldson IJ (2008). A novel mode of enhancer evolution: the Tal1 stem cell enhancer recruited a MIR element to specifically boost its activity.. Genome Res.

[21] Milat F, Ng KW (2009). Is Wnt signalling the final common pathway leading to bone formation?. Mol Cell Endocrinol.

[22] Bennett CN, Longo KA, Wright WS, Suva LJ, Lane TF (2005). Regulation of osteoblastogenesis and bone mass by Wnt10b.. Proc Natl Acad Sci U S A.

[23] Holmen SL, Giambernardi TA, Zylstra CR, Buckner-Berghuis BD, Resau JH (2004). Decreased BMD and limb deformities in mice carrying mutations in both Lrp5 and Lrp6.. J Bone Miner Res.

[24] Kato M, Patel MS, Levasseur R, Lobov I, Chang BH (2002). Cbfa1-independent decrease in osteoblast proliferation, osteopenia, and persistent embryonic eye vascularization in mice deficient in Lrp5, a Wnt coreceptor.. J Cell Biol.

[25] Takada I, Mihara M, Suzawa M, Ohtake F, Kobayashi S (2007). A histone lysine methyltransferase activated by non-canonical Wnt signalling suppresses PPAR-gamma transactivation.. Nat Cell Biol.

[26] Bodine PV, Zhao W, Kharode YP, Bex FJ, Lambert AJ (2004). The Wnt antagonist secreted frizzled-related protein-1 is a negative regulator of trabecular bone formation in adult mice.. Mol Endocrinol.

[27] Morvan F, Boulukos K, Clement-Lacroix P, Roman Roman S, Suc-Royer I (2006). Deletion of a single allele of the Dkk1 gene leads to an increase in bone formation and bone mass.. J Bone Miner Res.

[28] Lee NK, Sowa H, Hinoi E, Ferron M, Ahn JD (2007). Endocrine regulation of energy metabolism by the skeleton.. Cell.

[29] Ferron M, Wei J, Yoshizawa T, Del Fattore A, DePinho RA (2010). Insulin signaling in osteoblasts integrates bone remodeling and energy metabolism.. Cell.

[30] Heppner KM, Tong J, Kirchner H, Nass R, Tschop MH (2011). The ghrelin O-acyltransferase-ghrelin system: a novel regulator of glucose metabolism.. Curr Opin Endocrinol Diabetes Obes.

[31] Inui A (2001). Ghrelin: an orexigenic and somatotrophic signal from the stomach.. Nat Rev Neurosci.

[32] Prokunina-Olsson L, Hall JL (2010). Evidence for neuroendocrine function of a unique splicing form of TCF7L2 in human brain, islets and gut.. Diabetologia.

[33] De Ferrari GV, Moon RT (2006). The ups and downs of Wnt signaling in prevalent neurological disorders.. Oncogene.

[34] Hansen T, Ingason A, Djurovic S, Melle I, Fenger M (2011). At-Risk Variant in TCF7L2 for Type II Diabetes Increases Risk of Schizophrenia.. Biol Psychiatry.

[35] Savic D, Distler MG, Sokoloff G, Shanahan NA, Dulawa SC (2011). Modulation of Tcf7l2 expression alters behavior in mice.. PLoS One.

[36] Gough SC, ODonovan MC (2005). Clustering of metabolic comorbidity in schizophrenia: a genetic contribution?. J Psychopharmacol.

[37] Lin PI, Shuldiner AR (2010). Rethinking the genetic basis for comorbidity of schizophrenia and type 2 diabetes.. Schizophr Res.

[38] Risch N, Merikangas K (1996). The future of genetic studies of complex human diseases.. Science.

[39] Chakravarti A (1999). Population genetics – making sense out of sequence.. Nat Genet.

[40] Reich DE, Lander ES (2001). On the allelic spectrum of human disease.. Trends Genet.

[41] Folsom AR, Pankow JS, Peacock JM, Bielinski SJ, Heiss G (2008). Variation in TCF7L2 and increased risk of colon cancer: the Atherosclerosis Risk in Communities (ARIC) Study.. Diabetes Care.

[42] Sousa AG, Marquezine GF, Lemos PA, Martinez E, Lopes N (2009). TCF7L2 polymorphism rs7903146 is associated with coronary artery disease severity and mortality.. PLoS One.

[43] ECR genome browser. Available: http://ecrbrowser.dcode.org. Accessed 3 January 2012

