# Supplementary figures and images for: An in vivo cis-Regulatory Screen at the Type 2 Diabetes Associated TCF7L2 Locus Identifies Multiple Tissue-Specific Enhancers
Source: PLoS One. 2012 May 10;7(5):e36501. doi: 10.1371/journal.pone.0036501 (PMC3349716; doi:10.1371/journal.pone.0036501)

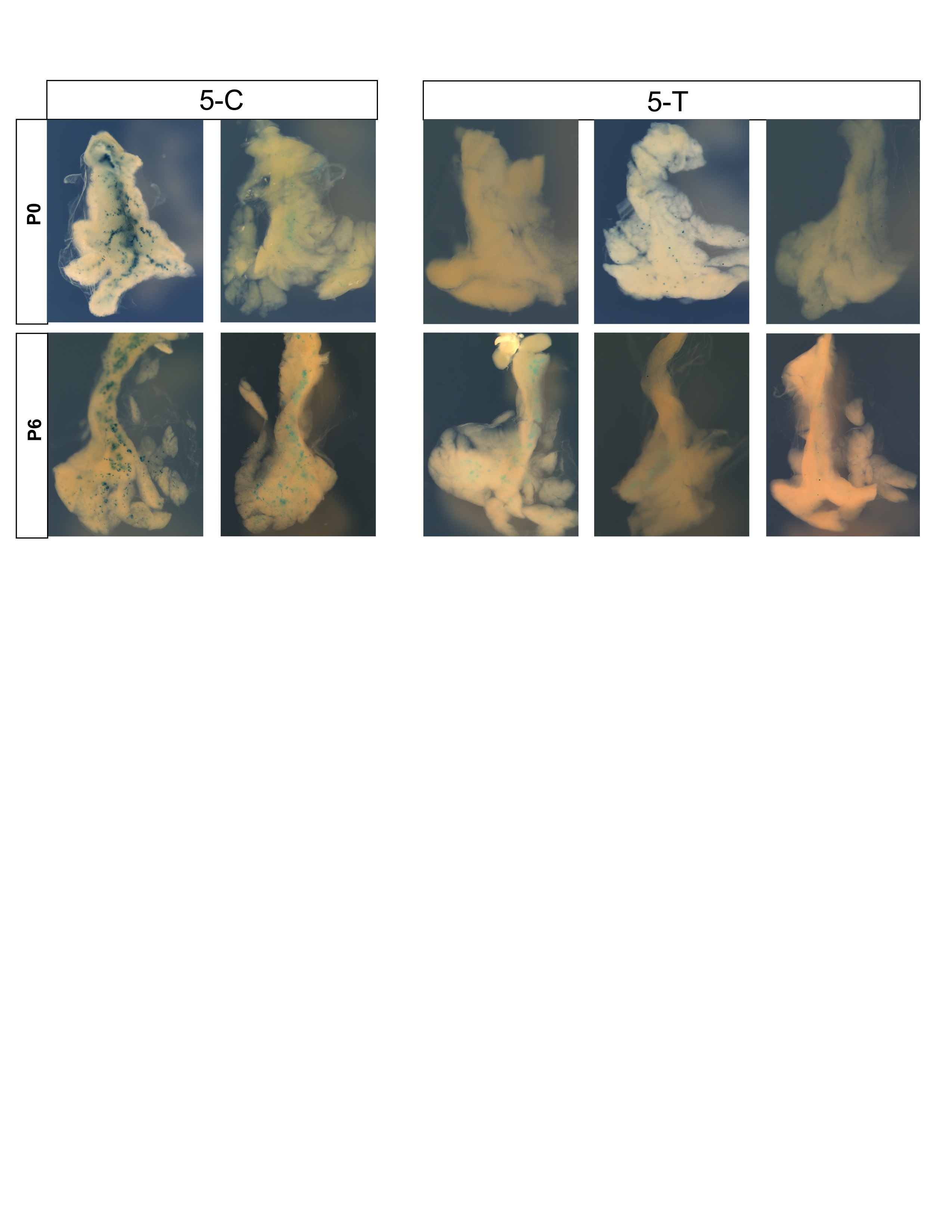

Supplement: Figure S1 — Postnatal analyses of pancreatic expression. Stable transgenic lines were stained for pancreatic beta-galactosidase activity on postnatal days 0 (P0, top panel) and 6 (P6, bottom panel). Pancreatic images for sequences spanning the protective C allele at SNP rs7903146 (5-C) and risk T allele at SNP rs7903146 (5-T) are shown. (TIFF) [file pone.0036501.s001.tiff]

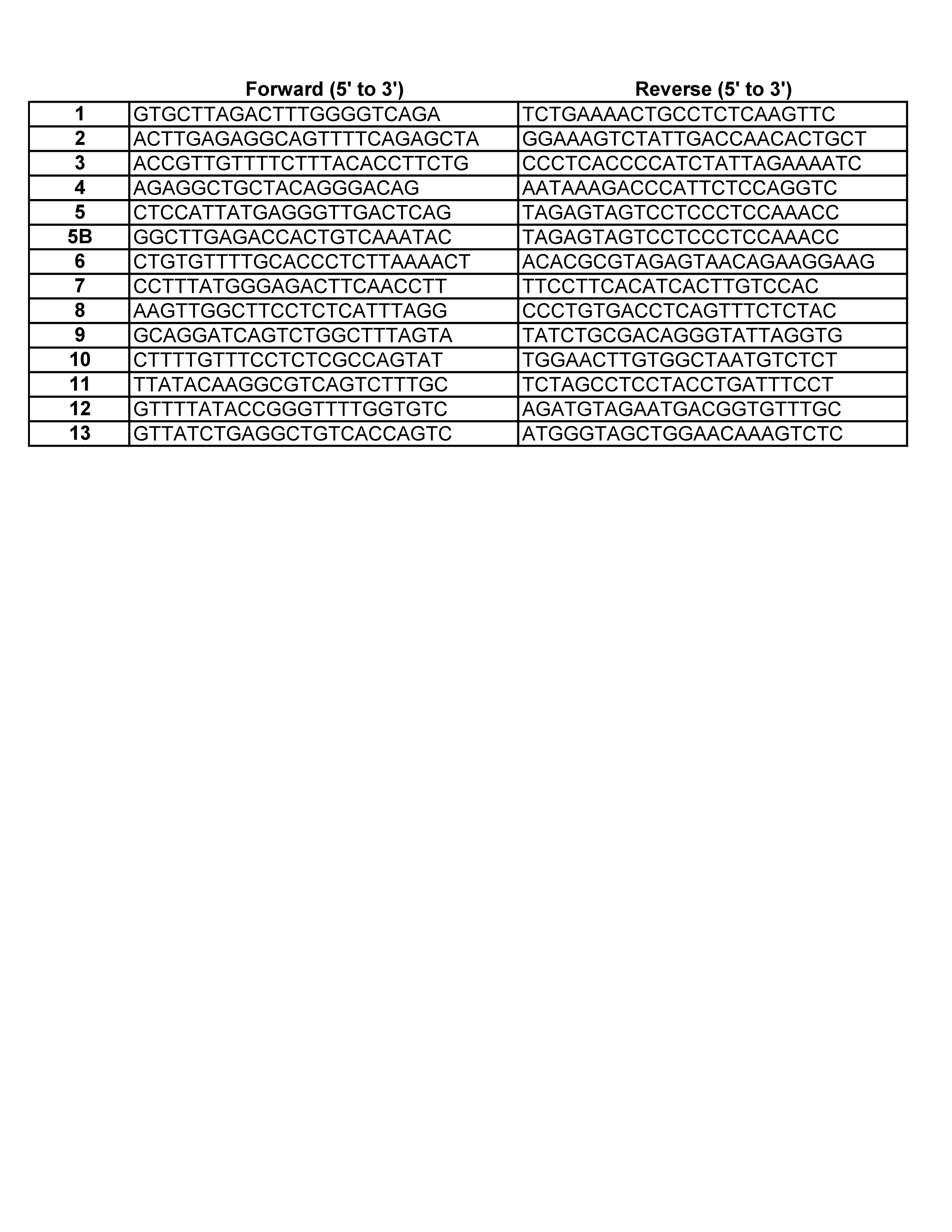

Supplement: Table S1 — Primer sequences for amplification of evolutionary conserved regions within the TCF7L2 association interval. The evolutionary conserved region (ECR) is numbered in the first column. Subsequent columns give the primer pair sequences (in 5′ to 3′ orientation) for each ECR. (TIFF) [file pone.0036501.s002.tiff]
